# Comprehensive proteome analyses of lysine acetylation in tea leaves by sensing nitrogen nutrition

**DOI:** 10.1186/s12864-018-5250-4

**Published:** 2018-11-26

**Authors:** Jutang Jiang, Zhongshuai Gai, Yu Wang, Kai Fan, Litao Sun, Hui Wang, Zhaotang Ding

**Affiliations:** 1 Tea Research Institute, Qingdao Agricultural University, 700 Changcheng road, Qingdao, 266109 Shandong China; 2 College of Life Science, Yantai University, Yantai, Shandong China; 3 Rizhao Tea Research Institute of Shandong, Rizhao, Shandong China

**Keywords:** *Camellia sinensis* (*L.*) O. Kuntze, Lysine acetylation, Acetylome, Metabolism, PTM, Nitrogen

## Abstract

**Background:**

*N*^ε^-Acetylation of lysine residues, a frequently occurring post-translational modification, plays important functions in regulating physiology and metabolism. However, the information of global overview of protein acetylome under nitrogen-starvation/resupply in tea (*Camellia sinensis*) leaves was limited. And the full function of lysine acetylated proteins of tea plants in nitrogen absorption and assimilation remains unclear.

**Results:**

Here, we performed the global review of lysine acetylome in tea leaves under nitrogen (N)-starvation/resupply, using peptide prefractionation, immunoaffinity enrichment, and coupling with high sensitive LC-MS/MS combined with affinity purification analysis. Altogether, 2229 lysine acetylation sites on 1286 proteins were identified, of which 16 conserved motifs in E^*^K^ac^K, K^ac*^K, K^ac*^R, K^ac*^HK, K^ac*^N, K^ac*^S, K^ac*^T, K^ac*^D, were extracted from 2180 acetylated peptides. Approximately, 36.76% of the acetylated lysines were located in the regions of ordered secondary structures. The most of the identified lysine acetylation proteins were located in the chloroplast (39%) and cytoplasm (29%). The largest group of acetylated proteins consisted of many enzymes, such as ATP synthase, ribosomal proteins and malate dehydrogenase [NADP], which were related to metabolism (38%) in the biological process. These acetylated proteins were mainly enriched in three primary protein complexes of photosynthesis: photosystem I, photosystem II and the cytochrome b6/f complex. And some acetylated proteins related to glycolysis and secondary metabolite biosynthesis were increased/decreased under N-resupply. Moreover, the PPI (protein-protein interaction) analysis revealed that the diverse interactions of identified acetylated proteins mainly involved in photosynthesis and ribosome.

**Conclusion:**

The results suggested that lysine acetylated proteins might play regulating roles in metabolic process in tea leaves. The critical regulatory roles mainly involved in diverse aspects of metabolic processes, especially in photosynthesis, glycolysis and secondary metabolism. A lot of proteins related to the photosynthesis and glycolysis were found to be acetylated, including LHCA1, LHCA3, LHCB6, psaE, psaD, psaN, GAPDH, PEPC, ENL and petC. And some proteins related to flavonoids were also found to be acetylated, including PAL, DFR, naringenin 3-dioxygenase and CHI. The provided data may serve as important resources for exploring the physiological, biochemical, and genetic role of lysine acetylation in tea plants. Data are available via ProteomeXchange with identifier PXD008931.

**Electronic supplementary material:**

The online version of this article (10.1186/s12864-018-5250-4) contains supplementary material, which is available to authorized users.

## Background

As one of the most common post-translational modifications (PTMS) to proteins, lysine acetylation is widely involved in such cellular biological activities as cell growth, apoptosis, cytokinetics and cell metabolisms [1, 2]. In recent years, a great deal of lysine-acetylated proteins had been discovered in microorganisms and mammalians [3]. Previous studies indicated that protein acetylation regulates a wide variety of important cellular processes, such as enzymatic activity [4, 5], protein interactions [2], protein stability [6] and metabolic pathways. However, in comparison with these organisms, the acetylome in tea plants by sensing nitrogen (N) nutrition is poorly studied [7].

Nitrogen (N) is the most important nutrient for tea plants. During the past decades, remarkable progress has been made in the basic understanding of N absorption, transportation and assimilation. In order to further study the acetylome of tea plants under N-starvation/resupply, we adopted an integrated system by using peptide prefractionation, immunoaffinity enrichment, and coupling with high sensitive mass spectrometry combined with affinity purification analysis. Based on our analysis of various biological processes, we identified 2229 acetylated sites on 1286 proteins in tea plants. These lysine acetylated proteins were localized in multiple compartments, such as nucleus, mitochondrion, cytoskeleton and so on. As far as we know, this is the first systematic study of lysine acetylome in tea leaves under N-starvation/resupply. Taken together, this study not only greatly improved our understanding of acetylation, but also greatly helps for us to further study the functions of lysine acetylation in tea plants.

## Methods

### Plant materials

One-year-old tea seedling of *Camellia sinensis* cv. QN3 hydroponically cultivated with the Full Nutrient (FN) solution in an air-conditioned chamber at 25 ± 2 °C/15 ± 2 °C (16 h day/8 h night), 80 ± 5% relative humidity and 280 μM•m^− 2^•s^− 1^ photon flux density 16 h light. The nutrient solutions were continuously aerated with an air pump in each hydroponic box. After 7 days the FN solution was replaced with another fresh FN solution, whereas the 0.84 mM (NH_4_)_2_SO_4_ was removed, in which the tea plants were subjected to N starvation for 7 days. After the N starvation treatment, the tea plants were harvested as 0 N sample. At the same time all other tea plants were supplied with fresh nutrient solution, whereas the 0.84 mM (NH_4_)_2_SO_4_ was replaced with 2.52 mM (NH_4_)_2_SO_4_ in which plants were re-supplied with N. The tea plants were harvested at 3 h and 3d after N -resupply after the harvest, the tea plants (tea leaves) were washed thrice with distilled water and finally with deionized water [8, 9].

### Reagents

All reagents unless otherwise stated were purchased from Sigma (St. Louis, America), such as Iodoacetamide (IAM), Nicotinamide (NAM) *et.al*. Acetonitrile (ACN) and TMT Kit (6 plex) were purchased from ThermoFisher Scientific (Waltham, USA). The lot number of lysine antibody agarose beads (PTM-104) were purchased from PTM Biolabs (Hangzhou, China). And the more detailed information was shown in Additional file 1: Table S1.

### Leaf maximum photochemical quantum yield and elemental analysis

Measurement of leaf maximum photochemical quantum yield was conducted on the leaves with a portable pulse amplitude modulation fluorimeter (PAM 2000; Heinz Walz GmbH, Effeltrich, Germany). The leaf maximum photochemical quantum yield of the tea leaves in the dark was monitored for approximately 20 min at 25°Cof saturating light, and the maximum quantum yield of PSII (*Fv/Fm*) was determined after the fluorescence had reached a steady level.

Samples were dried in an oven (ShangHai Fuma Test Equipment CO., LTD.). The dried samples were grinded to powder with a grinder. 0.5 g powder of each sample was put into a dry, clean PTFE vessel, and then 10 ml of nitric acid and 2 ml of perchloric acid were added and mixed. The solution was adjusted up to 25 ml with deionized water and then analyzed by inductively coupled plasma-optical emission spectroscopy (ICP-OES-Optima 8 × 00) (PerkinElmer, Inc., USA).

### Protein extraction, trypsin digestion and HPLC fractionation

The protein extraction of the tea leaves was performed according to previous reports [10]. The supernatant was reduced with 5 mM DTT for 30 min at 56 °C and alkylated with 11 mM IAM for 15 min at room temperature in darkness. The sample was then dissolved in 0.1 M TEAB. The protein was then digested with trypsin (Promega) at a trypsin-to-protein ratio of 1:50 for 12 h and then additional trypsin at 1:100 was added, and the mixture was incubated for an additional 4 h. After trypsin digestion, peptides were desalted by Strata X C18 SPE column (Phenomenex) and vacuum-dried. Then peptides were labeled with TMT kit according to the protocol of the manufacture [11]. Trypsin peptides were next fractionated by high pH reverse-phase HPLC using Thermo Betasil C18 column. The more detailed information was shown in Additional file 2.

### Affinity enrichment and LC-MS/MS analysis

The affinity enrichment was performed according to the former reports [12]. Briefly, tryptic peptides were firstly re-dissolved in NETN buffer and incubated with anti-lysine antibody beads (PTM Biolab) at 4 °C overnight with gentle end-to-end rotation. After that, the beads were washed four times with NETN buffer and twice with purified water. The bound peptides were eluted with 0.1% TFA. Finally, eluted peptides were cleaned with C18 ZipTips (Millipore) in accordance with the manufacturer’s instructions followed by HPLC/MS/MS analysis. And the LC-MS/MS analysis was performed according to previous report [13]. The more detailed information was shown in Additional file 2.

### Database search

The resulting MS/MS data were processed according to J C, M M and Cox J (v.1.5.2.8) [14, 15]. Searched for tandem mass spectra in a tea database. Trypsin/P was specified as cleavage enzyme allowing up to 4 missing cleavages. First search range was set to 5 ppm for precursor ions, main search range set to 5 ppm and 0.02 Da for fragment ions. Franklin Roosevelt was adjusted to < 1%, and the minimum value of the modified peptide was set > 40 [16]. All the detailed information was shown in the Additional file 2.

### Bioinformatics methods

The quantification of the lysine acetylated peptides and proteins were identified according to the TMT reporter [17, 18]. According to the acetylation quantification analysis, 1.5-fold change was set. The databases and softwares for bioinformatics analysis were shown in Additional file 2. When performing the bioinformatics analysis, *p*-value < 0.05 was considered significant according to the previous study [19–22].

### Western blotting

Proteins were separated by sodium dodecyl sulfate polyacrylamide gel electrophoresis (SDS-PAGE), then transferred to PVDF (Millipore) membranes and probed using anti-acetyl-lysine in the 1:1000 dilution (PTM Biolabs, Hangzhou, China). Secondary, the membranes were incubated in a 1:10000 dilution with horseradish peroxidase-conjugated antibody (Sigma).

## Results

### Detection of **lysine acetylated proteins in tea leaves under different N treatments**

**Fig. 1:**
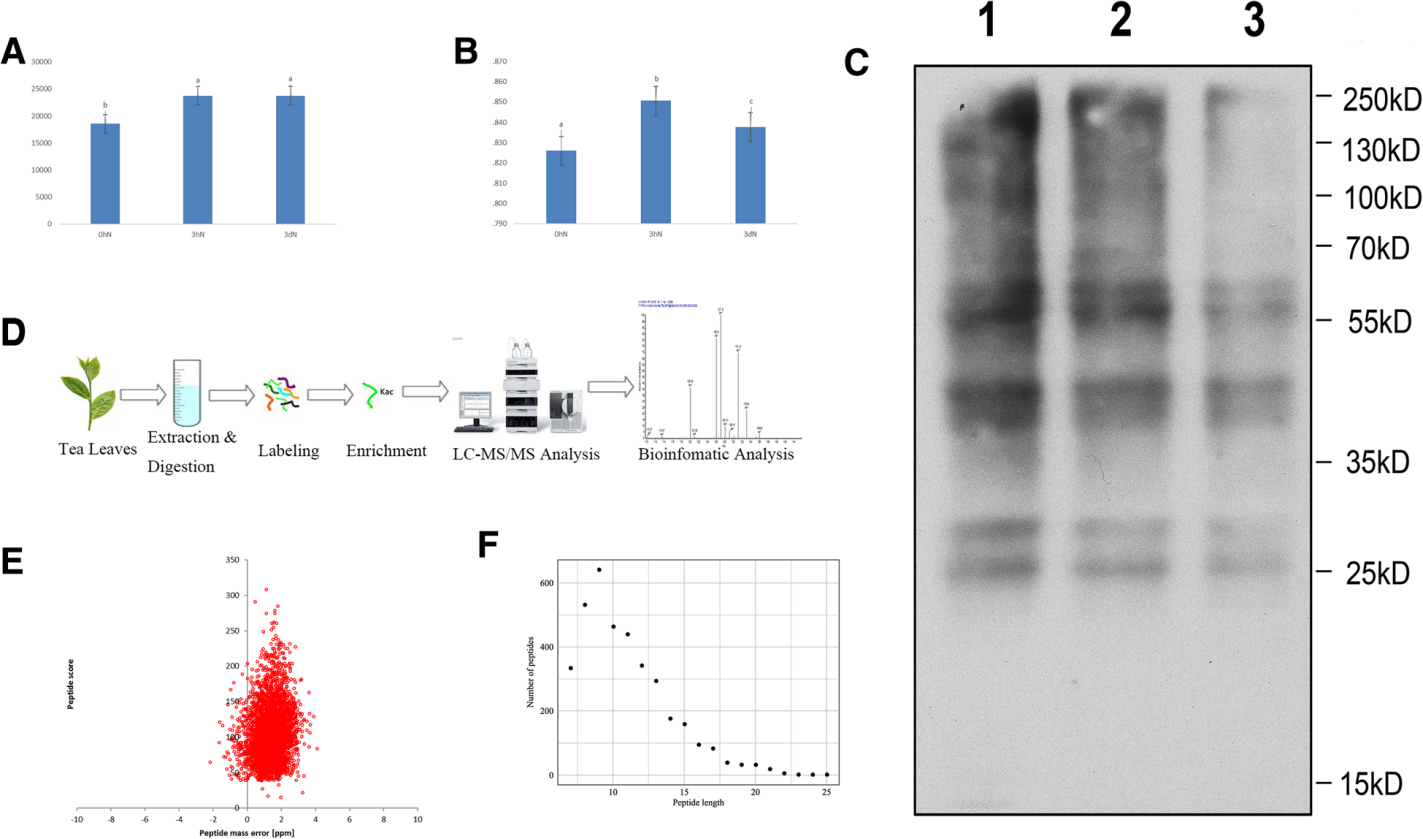
Detection of lysine acetylated proteins in tea leaves. **a** N contents in the tea leaves, μg·kg^− 1^ DW *n* = 3; **b** Chlorophyll fluorescence parameter, *n* = 4; Bars (a, b or c) indicate mean values ± standard errors, *p* < 0.05. **c** Western blotting of tea leaves proteins with anti-acetyllysine. 1lanes: 0 N acetylated proteins; 2lanes: 3hN acetylated proteins; 3lanes: 3dN acetylated proteins. 0 N indicates N starvation; 3hN and 3dN indicates 3 h and 3 days after N -resupply, respectively. **d** The workflow of integrated strategy for global mapping of lysine acetylation in tea leaves. **e** Mass error distribution of all identified peptides. **f** Peptide length distribution

To assess the physiological changes of the tea leaves under N treatments, we mainly detected the N contents of tea leaves and the leaf maximum photochemical quantum yield of PS II (*Fv/Fm*). Under N-starvation/resupply, the contents of N in tea leaves changed significantly from 0 N to 3hN (Fig. 1a). The contents of N in tea leaves under N-resupply increased by 27.8% from 0 N to 3hN, but then kept stable from 3hN to 3dN. The leaf maximum photochemical quantum yield of PS II (*Fv/Fm*) of different treatment groups were significantly influenced by the N contents of tea leaves. Under 3 h N-resupply, *Fv/Fm* was higher than that of N starvation or 3d N-resupply. And *Fv/Fm* of 3d N-resupply was higher than that of N starvation (Fig. 1b**,** Additional file 3: Table S2). In order to research overall acetylated proteins in tea leaves under N treatments, the Western Blotting assay was performed with protein extracts from tea leaves using anti-acetyl-lysine antibody. The results indicated that the multiple lysine-acetylated protein bands of different N treatments were detected and showed stronger reactions to the anti-acetyl-lysine **(**Fig. 1c). So, protein acetylation occurred in tea leaves under N-starvation/resupply and lysine-acetylated peptides can be affinity enriched for further identification and analysis.

**Fig. 2:**
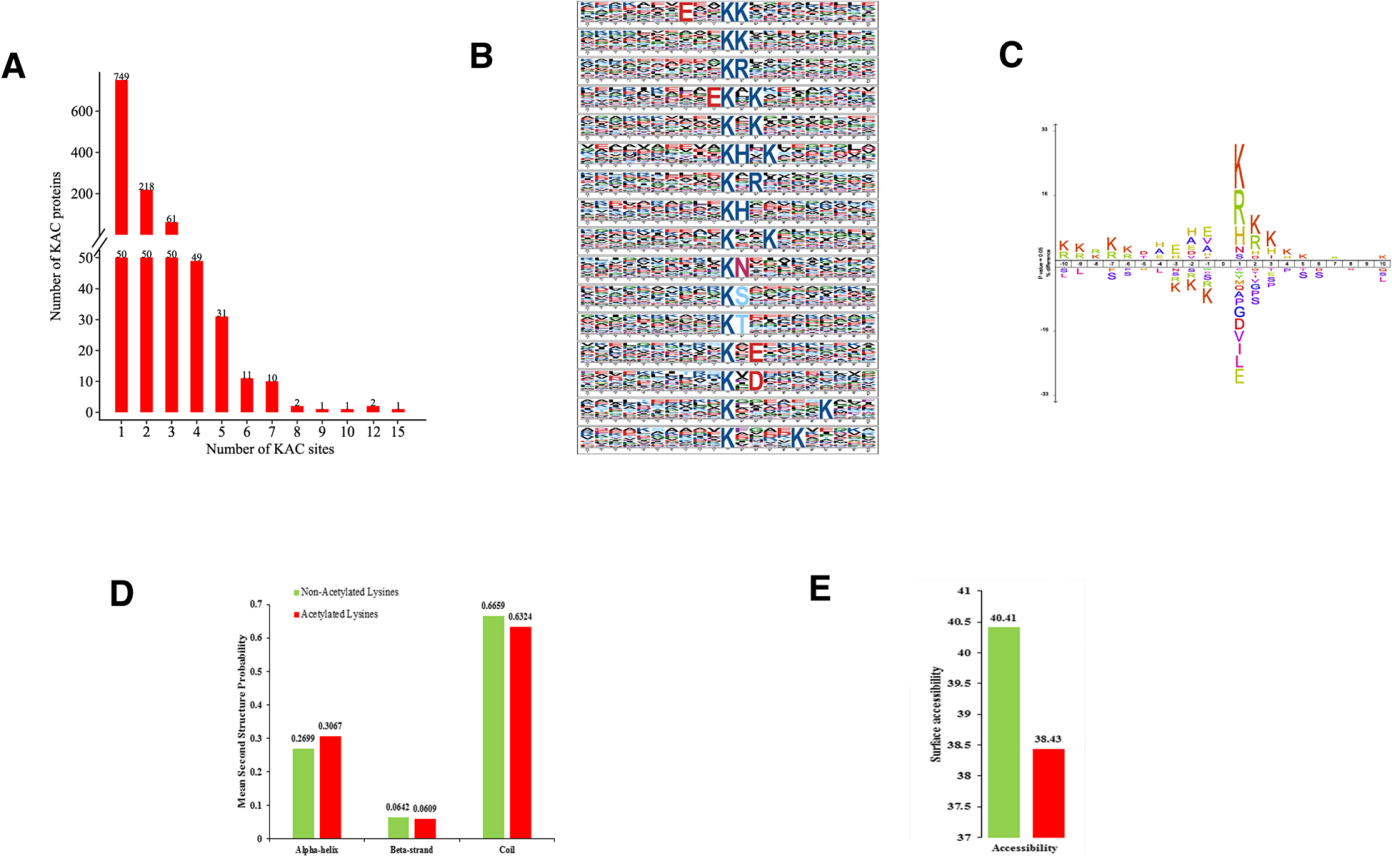
Motif analysis of lysine-acetylated peptides. **a** Distribution of acetylated proteins based on the numbers of acetylation sites. **b** Sequence probability logos of significantly enriched acetylation site motifs for±10 amino acids around the lysine acetylation sites. **c** Heat map reflects the relative frequencies of amino acids in specific positions. **d** Distribution of secondary structures containing lysine acetylation sites. **e** Predicted surface accessibility of acetylation sites

The workflow of experimental procedures used in the study was shown in Fig. 2d. In order to verify the validity of MS data, the quality errors of all confirmed acetylpeptides were checked. The distribution of quality errors was close to zero, and the length of most acetylation peptides was distributed between 7 and 25 (Fig. 2e, f). This confirmed the high accuracy of MS data. In total, 1286 proteins were shown to be acetylated, with 2229 unique acetylated sites in tea leaves under N-starvation/resupply (Additional file 4: Table S3). Up to now, tea leaves had a great quantity of acetylated proteins among the plants reported, reflecting a potentially vital role of this modification in tea plants, which has drawn our attention.

### Distribution and motif analysis of lysine acetylation sites

To assess the distribution of acetylation sites on the proteins of tea leaves, we calculated the numbers of modified sites on the acetylated proteins. The average degree of acetylation was 1.7 per protein. The results indicated that 62.1% (799/1286) contained a single acetylated site, 20.8% (268/1286) contained two acetylated sites, and 8.6% (111/1286) contained three acetylated sites (Fig. 2a). It is noteworthy that 59 proteins contained five or more acetylated sites and 4 had at least 10 sites, such as m.49400 contained 10 acetylated sites, m.1156 and m.12353 contained 12 acetylated sites, m.45083 contained 15 acetylated sites (Additional file 5: Table S4). According to the protein ratio results, we found that the numbers of proteins were similarity in the different ratios, but the numbers of proteins located in the 0 were reduced with N-resupply. The longer time for the N-resupply, the numbers of proteins were reduced more significantly (Additional file 6: Figure S1).

To further evaluate the natural properties of acetylated lysines in tea, we investigated the motifs of all identified lysine residues using Motif-x program. A total of 16 conserved motifs in E^*^K^ac^K, K^ac*^K, K^ac*^R, K^ac*^HK, K^ac*^N, K^ac*^S, K^ac*^T, K^ac*^D, were extracted from 2180 acetylated peptides. The most common combination was K^ac^*K, which was represented by 343 (17.4%) of the enrichment motifs. Among these motifs, three distinct types of residues were located upstream/downstream of the acetylated lysine: three positively charged (basic) residues, including lysine (K), arginine (R) or histidine (H), and the two negatively charged residues, including glutamic acid (E) or aspartic acid (D), and two residues with a hydroxyl group, including serine (S) and threonine (T), which were enriched at the + 1 position on the C-terminus side (Fig. 2b**,** Additional file 7: Table S5, Additional file 8: Table S6).

With respect to the general amino acid composition around an acetylated lysine site, Ice Logo heat maps were used to assess whether specific amino acids were significantly enriched or depleted by identifying the relative frequencies of the amino acids at specific positions surrounding the acetylated site (10 amino acids upstream and 10 amino acids downstream from the modification sites). The amino acid frequencies determined with the heat map were consistent by those determined with Web Logo (Fig. 2c). These amino acids could be divided into two categories: the + 1, + 2 or + 3 positions, which were alkaline residues with long side chains (H, K or R), and the − 1 or − 3 positions, which were residues with long hydrophobic side chains (W, V or A). These results showed that amino acid residues with alkaline and hydrophobic side chains might play an important role in acetylation. These new residues of amino acids in tea leaves would potentially provide acetylated binding sites for future studies.

To understand the local secondary structures in more details, we compared the secondary structures surrounding the acetylated lysines with those surrounding all lysines using the NetSurfP software. Approximately, 36.76% of the acetylated lysines were located in the regions of ordered secondary structures. Among them, 30.67% sites were located in α-helices and 6.09% sites in β-strands. The remaining 63.24% were located in disordered regions of the proteins (Fig. 2d). Nevertheless, it seems that the proteins in tea leaves had no tendency of acetylation according to the similar of distribution patterns between the acetylated lysines and non-acetylated lysines. In addition, we evaluated the surface accessibility of acetylated lysine sites, too. The data indicated that 38.43% of the acetylated lysine sites were exposed to the protein surface, compared with 40.41% of non-acetylated lysine residues (Fig. 2e). Consequently, slight change might be happened in the surface property of proteins because of lysine acetylation.

### Functional characterization and subcellular localization of lysine acetylated proteins in tea leaves

**Fig. 3:**
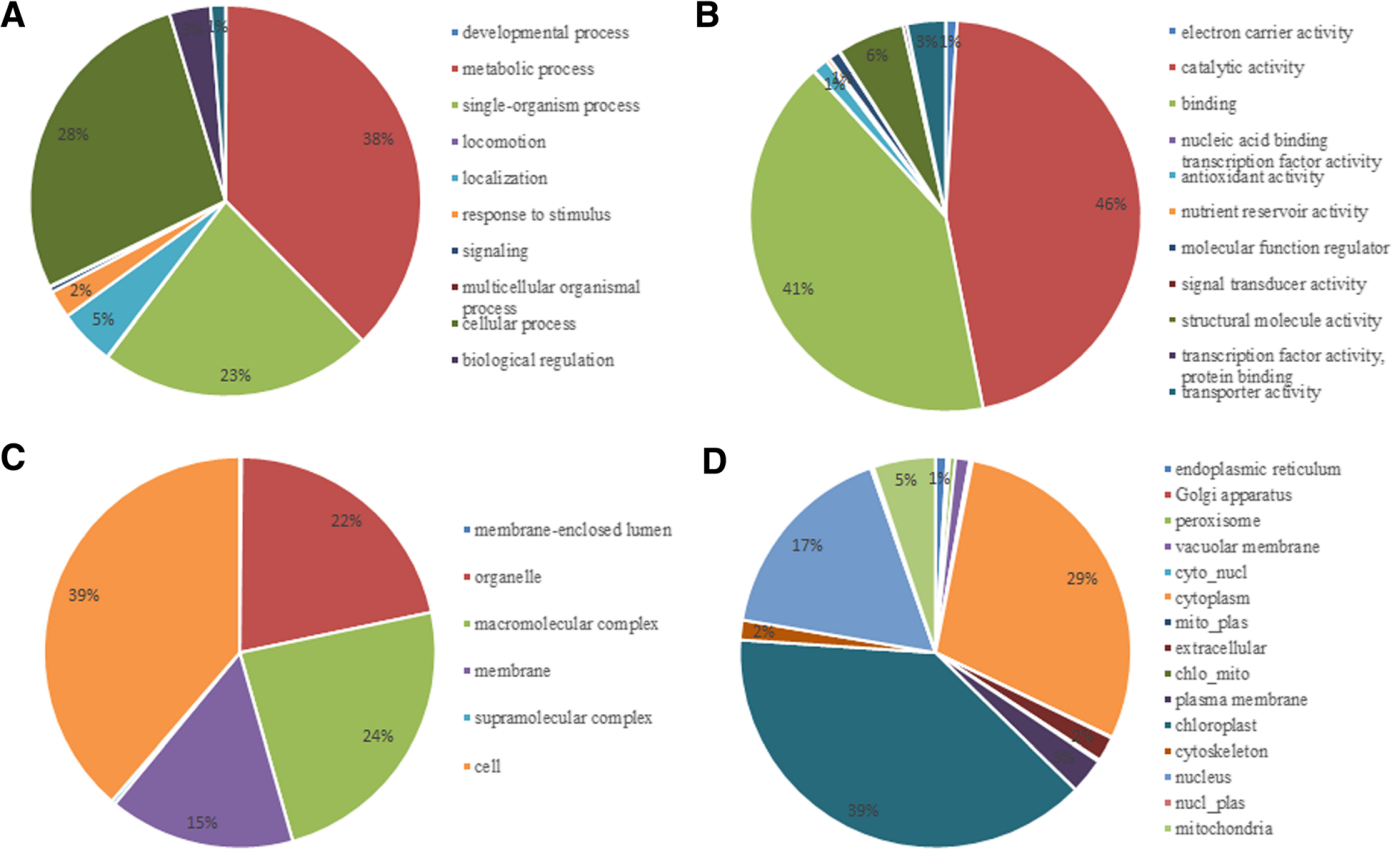
Functional classification of acetylated proteins in tea. **a** Classification of the acetylated proteins based on biological process. **b** Classification of the acetylated proteins based on molecular function. **c** Classification of the acetylated proteins based on cellular component. **d** Subcellular localization of the acetylated proteins

GO functional classification of all the acetylated proteins was investigated based on their biological process, molecular function and cellular component so that we can further and better understand the acetylome in tea plants. The results indicated that the largest group of acetylated proteins consisted of many enzymes, such as ATP synthase, ribosomal proteins and malate dehydrogenase [NADP], which were related to metabolism (38%) in the biological process (Fig. 3a). For the molecular function category **(**Fig. 3b), the acetylated proteins related to catalytic activity and binding functions were identified, accounting for 46 and 41% of all the acetylated proteins, respectively. Regarding the cellular component category **(**Fig. 3c), most of the acetylated proteins were related to cell (39%), macromolecular complex (24%) and organelle (22%). Further studies showed that the major classes were similarly under the different N treatments, but the numbers of proteins were different under the N-starvation/resupply. For instance, there were 82 acetylated proteins associated with metabolic process. Among them there were 27 acetylated proteins associated with photosynthesis and glycolysis in the 3hN/0 N. There were 80 and 29 acetylated proteins in the 3dN/0 N. And there were 90 and 21 acetylated proteins in the 3dN/3hN. The more detailed information was provided in the Additional file 9: Table S7.

A large proportion of the identified acetylated proteins in tea leaves were located to the cytoplasm (29%) and chloroplast (39%) which were shown in subcellular localization analysis. In further researches, we discovered that 10% of the acetylated proteins presented in chloroplast participated in the process of tricarboxylic acid (TCA) cycle and photosynthesis. As expected, 17% of acetylated proteins, including histones and nonhistones were located in the nucleus, confirming the regulatory role of lysine acetylation in post-transcriptional regulation. Beyond that, we found that some proteins were distributed in the mitochondria (5%), cytoskeleton (2%), plasma membrane (3%) and endoplasmic reticulum (1%) (Fig. 3d). Further studies showed that the numbers of acetylated proteins associated with photosynthesis were different under N-starvation/resupply. For example, there were 7 different acetylated proteins in the 3hN/0 N. There were 5 different acetylated proteins in the 3dN/0 N. And there were 11 different acetylated proteins in the 3dN/3hN. The results showed that the distribution of proteins was a dynamic process that related to metabolized continually and changed momentarily. These data, as well as the results of GO functional classification, showed that lysine acetylated proteins had extensive biological functions in tea leaves.

### The enrichment analysis of lysine-acetylated proteins in tea leaves under different N treatments

**Fig. 4:**
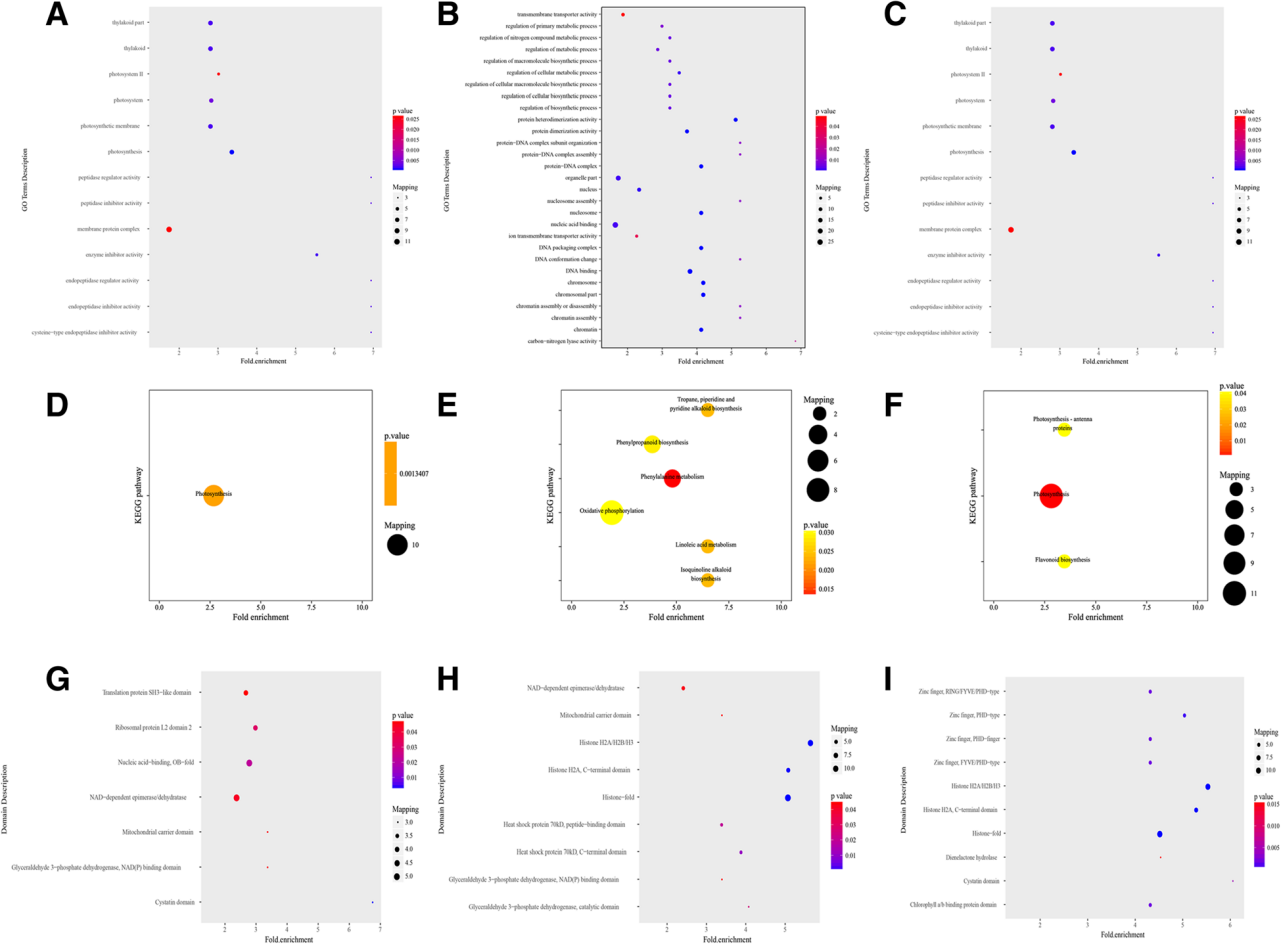
GO-based enrichment analysis of identified acetylated proteins. **a** GO-based enrichment analysis of 3hN/0 N. **b** GO-based enrichment analysis of 3dN/0 N. **c** GO-based enrichment analysis of 3dN/3hN. **d** KEGG pathway-based enrichment analysis of 3hN/0 N. **e** KEGG pathway-based enrichment analysis of 3dN/0 N. **f** KEGG pathway-based enrichment analysis of 3dN/3hN. **g** Protein domain enrichment analysis of 3hN/0 N. **h** Protein domain enrichment analysis of 3dN/0 N. **i** Protein domain enrichment analysis of 3dN/3hN

To complete which types of proteins are preferred targets for lysine acetylation, the analysis of GO, KEGG pathway and protein domains was successfully accomplished (Fig. 4**,** Additional file 10: Table S8).

GO enrichment analysis based on the biological process showed that photosynthesis, translation and metabolic process were enriched in acetylated proteins in the 3hN/0 N **(**Fig. 4a). Similarly, the photosynthesis ranked first in the 3dN/3hN (Fig. 4c), followed by the metabolic process and organelle activity. While the metabolic process ranked first in the 3dN/0 N (Fig. 4b), followed by the biosynthetic process. As far as the molecular function category was concerned, the activities of enzyme inhibitor and enzyme regulator were significantly enriched in the 3hN/0 N. However, DNA binding and protein activity were enriched in the 3dN/0 N and 3dN/3hN. There were a few differences among the three different ratios. Consistently, for the cellular components the acetylated proteins were significantly enriched in photosystem and thylakoid part in the 3hN/0 N. But the organelles and DNA complexes were significantly enriched in the 3dN/0 N and 3dN/3hN.

In order to better understand its general functions in tea leaves, these acetylated proteins were mapped to KEGG metabolic pathways. The results showed that the KEGG pathway of the photosynthesis was enriched significantly in the 3hN/0 N and 3dN/3hN (Fig. 4d, f). The metabolic process and biosynthesis were enriched significantly in the 3dN/0 N (Fig. 4e). This indicated that lysine acetylation occurred on many proteins related to photosynthesis in the 3hN/0 N. Meanwhile, the enrichment proteins took part in photosynthesis and flavonoid biosynthesis in the 3dN/3hN. But most acetylated proteins were related to amino acid metabolism and biosynthesis, such as phenylalanine metabolism, linoleic acid metabolism and phenylalanine biosynthesis in the 3dN/0 N. Furthermore, the enzymatic activity and the NAD (P)-binding domain were enriched notably in tea leaves under the N-starvation/resupply.

### The analysis of interaction network in lysine acetylated proteins

For the purpose of deeply understanding how these acetylated proteins are related and how the acetylated proteins involved in different pathways crosslink to each other, we chose STRING database and Cytoscape software (https://string-db.org) to construct the PPI (protein-protein interaction) networks for the distinct proteins. We extracted several highly rich interactive clusters from the entire interaction network by means of the MCODE plug-in tool kit.

**Fig. 5:**
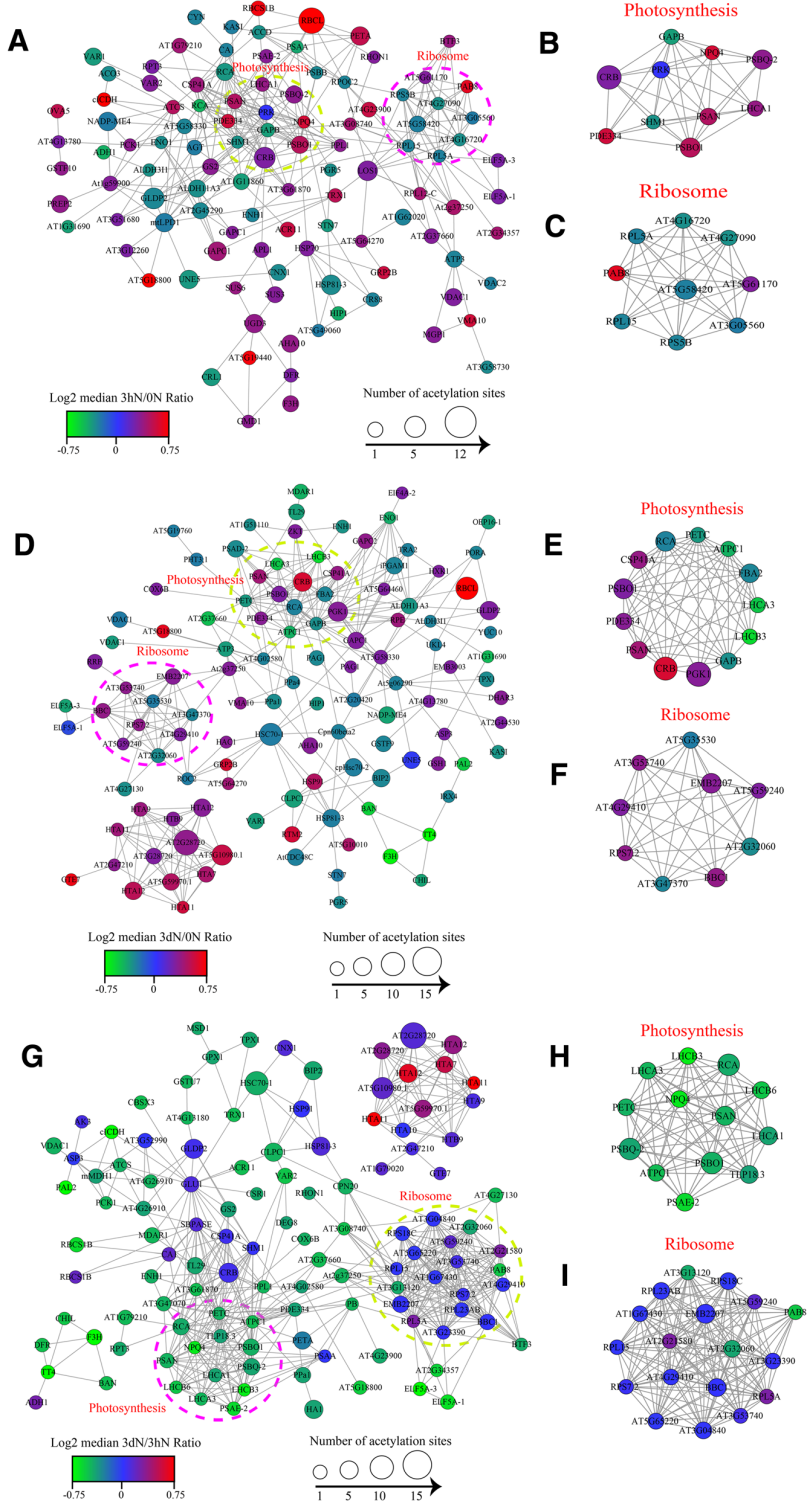
Interaction networks of the acetylated proteins. **a** the whole PPI network of 3hN/0 N. **b** Interaction network of acetylated proteins involved in photosynthesis of 3hN/0 N. **c** Interaction network of acetylated proteins associated with ribosome of 3hN/0 N. **d** the whole PPI network of 3dN/0 N. **e** Interaction network of acetylated proteins involved in photosynthesis of 3dN/0 N. **f** Interaction network of acetylated proteins involved in photosynthesis of 3dN/0 N. **g** the whole PPI network of 3dN/3hN. **h** Interaction network of acetylated proteins involved in photosynthesis of 3dN/3hN. **i** Interaction network of acetylated proteins associated with ribosome of 3dN/3hN

**Fig. 6:**
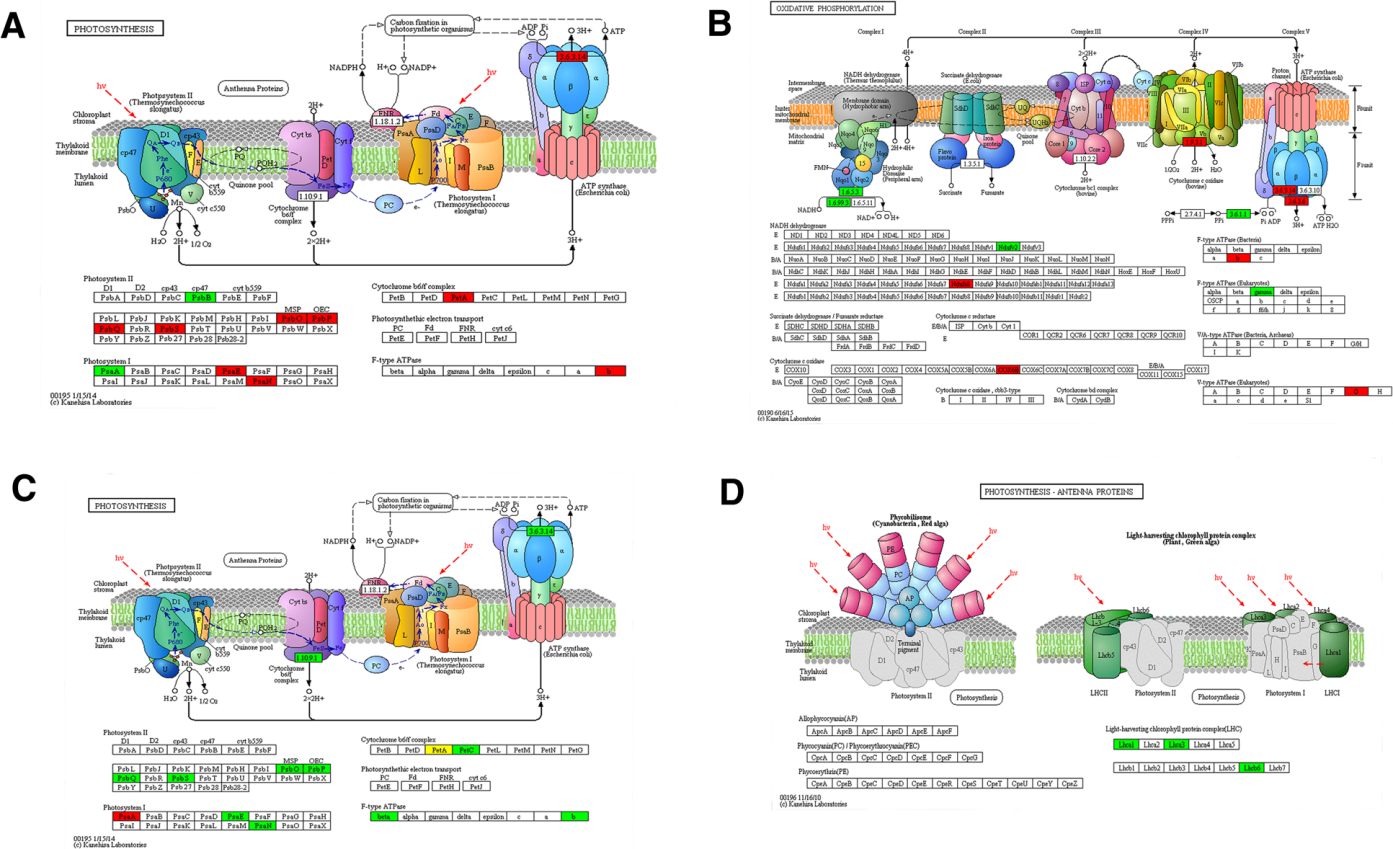
Representative significantly enriched photosynthesis related KEGG pathways. **a** the 3hNvs0N significantly enriched photosynthesis pathway **b** the 3dNvs0N significantly enriched oxidative phosphorylation pathway **c, d** the 3dNvs3hN significantly enriched photosynthesis pathway. Red color indicates up-regulated acetylated proteins in the pathway; Green color indicates down-regulated acetylated proteins; Yellow color indicates the up/down-regulated acetylated proteins. The figures were come from the KEGG: new perspectives on genomes, pathways, diseases and drugs. Nucleic Acids Res. 45, D353-D361

Compared 3hN with 0 N, there were 108 acetylated proteins in interaction which mapped to the protein interaction database (Fig. 5a**,** Additional file 11: Table S9). Thereinto, 62 were up-regulated, including psaN, psbO, psbS, rbcL, rbcS and IDH1, and 46 were down-regulated, including GAPA, psbB, psaA, AGXT, accD and GLDC. They were clustered into 8 groups. The top group (Cluster I) consisted of photosynthesis-related proteins. These acetylated proteins could be roughly classified into chlorophyll a/b binding protein domain and NAD (P)-binding domain, of which 10 highly correlated acetylated proteins were retrieved, including GAPB, psbO, psbQ and PsaN **(**Fig. 5b**,** Additional file 12**:** Table S10). Whereas Cluster II consisted of the proteins involved in ribosome, of which 9 interconnected clusters of acetylated proteins were retrieved **(**Fig. 5c**,** Additional file 13: Table S11). In the Cluster I, the acetylated protein GAPB interacted with the acetylated ribosome protein RPL15. Meanwhile, numerous photosynthesis proteins interacted with GAPB, and many ribosome proteins interacted with RPL15. Additionally, in the enrichment analysis of KEGG pathway, several acetylated proteins concentrated in photosynthetic pathways and one typical pathway was shown in Fig. 6a. The almost all the core parts of photosynthesis, such as photosystems (I and II), cytochrome b6f complex, electron transports, and ATP synthases, were acetylated in several different subunits.

Compared 3dN with 0 N, there were 117 acetylated proteins in interaction which were mapped to the protein interaction database **(**Fig. 5d**,** Additional file 14: Table S12). Thereinto, 54 were up-regulated, including psaN, psbO, GAPDH, GLDC, COX6B and PGK, and 63 were down-regulated, including GAPB, petC, LHCA3, ANR, ENL and psaD. They were clustered into 8 groups. The top group (Cluster I) consisted of photosynthesis-associated proteins**,** in which 13 highly interconnected clusters of acetylated proteins were retrieved, including PGK, GAPB, petC, psaN and psbO **(**Fig. 5e**,** Additional file 15: Table S13). These acetylated proteins could be classified into three major groups according to their functions in photosynthesis, namely chlorophyll a/b binding proteins, NAD (P)-binding proteins and ATPase proteins. Whereas Clusters II consisted of proteins involved in ribosome **(**Fig. 5f**,** Additional file 16: Table S14), in which 9 interconnected acetylated proteins were retrieved. In this network, 20 lysine acetylated proteins were identified with the node degree over 10, of which PGK, GAPB, petC and ALDO had the highest degree. Among them, the acetylated protein GAPB had the highest degree and interacted with photosynthesis proteins. Meanwhile, numerous ATP binding proteins and many NAD (p) binding proteins interacted with GAPB. Moreover, in our KEGG pathway enrichment analysis, one representative pathway was shown in Fig. 6b. The almost all the core parts of oxidative phosphorylation, such as NADH dehydrogenase, Cytochrome c oxidase/reductase and ATPase, were acetylated in several individual subunits. There were a few differences from the 3hN/0 N.

Compared 3dN with 3hN, there were 118 acetylated proteins in interaction which were mapped to the protein interaction database **(**Fig. 5g**,** Additional file 17: Table S15). Thereinto, 46 were up-regulated, including psaN, psbO, GAPDH, GLDC, COX6B and PGK, and 72 were down-regulated, including GAPB, petC, LHCA3, ANR, ENO and psaD. They were also clustered into 8 groups. The top group (Cluster I) identified above consisted of ribosome-associated proteins, in which 18 highly interconnected acetylated proteins were retrieved **(**Fig. 5i**,** Additional file 18: Table S16)**.** Whereas Cluster II consisted of the proteins involved in photosynthesis, in which 13 interconnected acetylated proteins were retrieved, including psbQ, psbO, LHCB6, LHCA1 and LHCA3 **(**Fig. 5h**,** Additional file 19: Table S17). These acetylated proteins could be roughly attributed to chlorophyll a/b binding proteins. As shown in the Fig. 5g, 46 lysine acetylated proteins were identified with the node degree over 10, of which 11 were over 20 node degrees, of which subunit ribosomal protein, ATPase and petC had the highest degree. Among them, the acetylated protein petC had the highest degree and interacted with NAD (p)-binding proteins. Meanwhile, numerous ATP binding proteins and many primary metabolism proteins interacted with petC. In the KEGG pathway enrichment analysis, several pathways related to photosynthetic were enriched and two representative pathways were shown in Fig. 6c, d. The almost all the core parts of light reaction were acetylated in several different subunits, such as photosystems (I and II), cytochrome b6f complex, electron transports and ATP synthases, indicated that these lysine acetylated proteins had close relationship in photosystem. Besides, photosynthesis antenna proteins (LHCA1, LHCA3 and LHCB6) were also acetylated in lysine sites. It can be seen clearly that, almost all the acetylated proteins had close interaction in the photosynthesis and metabolism in tea leaves under N-starvation/resupply.

## Discussion

In this study, we determined the acetylome of tea leaves with the identification of 2229 lysine acetylation sites on 1286 proteins. This is the first catalog of acetylated proteins in tea leaves by sensing nitrogen nutrition. These modified proteins were involved in different metabolic processes and participated in a variety of biological processes. Importantly, a lot of proteins related to photosynthesis and glycolysis were found to be acetylated, some proteins related to flavonoids were also found to be acetylated. Furthermore, the analysis of protein interaction network demonstrated that a wide range of interactions were modulated by lysine acetylation.

With the results of our study, we found that 498 of acetylated proteins were located in chloroplast in tea leaves and many of them were involved in photosynthesis. According to the ratio results, the acetylated proteins associated with photosynthesis were found to be up/down-regulated in tea leaves under different N treatments, suggesting that the acetylated proteins located in chloroplast might play important roles in regulating photosynthesis. For example, after N-resupply, the chlorophyll a/b binding protein was significantly up-regulated, while NADP+ was down-regulated. According to the KEGG pathway analysis, the high expression of chlorophyll a/b binding protein in PSI may facilitate the photon capturing and then excite energy to reaction centers to increase NADPH generation. This was confirmed by the value of *Fv/Fm* in our study (Fig.1). Furthermore, multiple types of chlorophyll a/b binding proteins, including LHCA1, LHCA3 and LHCB6, were identified in tea leaves after N treatments. We found that LHCA1 was up-regulated under 3 h N-resupply. But the LHCA3 and LHCB6 were significantly down-regulated in the 3dN/0 N and 3dN/3hN. It is known that light-harvesting complexes (LHCs) are the major constituents of antenna systems in plants, which remove light energy to the chlorophyll a particle at the backlash center of photosystems [23]. For example, in wheat, 100 of acetylated proteins were localized in chloroplast and many of them were involved in photosynthesis, such as LHC a-binding protein (Lhca1) and LHC b-binding proteins (Lhcb3, Lhcb5 and Lhcb6) [24]. In the “Anjin Baicha”, the LHCA1, was identified several acetylated sites and associated with the periodic albino phenotype [25]. Thus, we could speculate that the reversible lysine acetylation might influence the function of antenna protein complex and maintain the photosynthesis rate in tea leaves after N-resupply.

PSII is a complete membrane protein complex containing more than 20 subunit proteins and numerous cofactors [26]. In this study, several subunits important for this course, including psbO, psbQ, psbS and psbP, were all identified as acetylated proteins in tea leaves under N-starvation/resupply. The electron transfer proteins were up-regulated in the 3hN/0 N, while the proteins were down-regulated in the 3dN/0 N and 3dN/3hN. It can be shown that PSII activity was increased by shorter N-resupply. In support of our findings, the electron transfer proteins were found to be lysine acetylated in Cyanobacterium *Synechocystis* sp. PCC 6803 and strawberry [27, 28]. In addition, PSI is a multisubunit complex with membrane embedded that can transfer light-induced electrons to soluble electron acceptors such as ferredoxin (Fd) [29]. In our study, four PSI subunits (PsaA, PsaE, PsaD, and PsaN) were also found to be acetylated in tea leaves. Among them, in the 3hN/0 N, the PsaA was down-regulated, while the PsaE and PsaN were up-regulated. But the trends of the three acetylated proteins were opposite in the 3dN/3hN. To our knowledge, there were no reports about the functions of the three proteins. Interestingly, the PsaD was down-regulated in the 3dN/0 N. And the *Fv/Fm* reduced from 3hN to 3hN. A former study in *Synechocystis* showed that PsaD was likely to work with many PSI subunits, and acetylated proteins affected the interaction of PsaD and Fd to regulate Fd activity [12]. We speculated that the reduction of photosynthesis had relationship with the PsaD. In addition, the acetylated protein petA was up-regulated in the 3 h N-resupply, but the cytochrome b6-f and COX6B were down-regulated in the 3d N-resupply. Similarly, some chloroplast ATP synthases were found to be changed in tea leaves under N-starvation/resupply. For example, the acetylated protein ATPF0B and ATPeV1G were up-regulated in the N-resupply, the ATPeF1G was down-regulated in the N-resupply. The previous study showed that the cytochrome complex could transfer electrons from PSII to the PSI, which affected the production of ATP and NADPH [30, 31]. We speculated that these acetylated proteins in tea leaves under N-starvation/resupply can transfer electrons from PSII to the PSI.

Glycolysis is a way of catabolic anaerobic, which produces pyruvate, the reductant and ATP, which provides the metabolites needed for anabolism [32]. Glycolysis is using for another bioenergy pathway in the organism under stress conditions [33]. In our study, we identified the 15 acetylated proteins in the glycolysis pathway, of which three enzymes, including ENL, gapN and ALDH, were down-regulated after N-resupply. The GAPDH was up-regulated in the 3hN/0 N and 3dN/0 N, while the PEPC was up-regulated in the 3hN/0 N, but down-regulated in the 3dN/3hN and 3dN/0 N. We also found that the numbers of different expression proteins in the TCA cycle changed significantly after N-resupply. And the ATP binding and NAD acetylated proteins were up-regulated in the 3hN/0 N, but ATP binding and MDH proteins were down-regulated in the 3dN/ 0 N and 3dN/3hN. Previous studies showed that GAPDH of higher plants catalyzed an NADPH-consuming reaction, which was part of the Calvin cycle. This reaction was regulated by light via thioredoxins and metabolites, while a minor NADH-dependent activity was constant and constitutive. Formation and dissociation of this supramolecular complex contributed to light-dependent modulation of both enzyme activities and hence to the overall regulation of photosynthetic metabolism. Bringing several glycolytic enzymes close to each other was expected to greatly increase the overall speed of glucose breakdown [34–36]. So, we speculated that the up-regulation of GAPDH might accelerate the degradation of glucose, which affected the production of NADPH in tea leaves under N-resupply. Enolase (ENL), also known as phosphopyruvate hydratase, is a metalloenzyme responsible for the catalysis of the conversion of 2-phosphoglycerate (2-PG) to phosphoenolpyruvate (PEP), the ninth and penultimate step of glycolysis [37]. In the present study, the down-regulation of acetylated ENL and PEPC showed that the acetylated ENL and PEPC might speed down the glycolysis of tea leaves under N-resupply. As for pasN, there is no report about its functions of acetylation in plants up to now. For this reason, it is necessary to carry out in-depth research on its functions in tea plants by sensing N nutrition.

Tea flavonoids play important roles in tea quality. Phenylalanine ammonia lyase (PAL) is the first and committed step in the phenyl propanoid pathway and is therefore involved in the biosynthesis of the polyphenol compounds, such as flavonoids, phenylpropanoids, and lignin in plants [38]. The activity of PAL is induced dramatically in response to various stimuli, such as tissue wounding, pathogenic attack, light, low temperatures, and hormones. PAL is involved in 5 metabolic pathways: tyrosine metabolism, phenylalanine metabolism, nitrogen metabolism, phenylpropanoid biosynthesis, and alkaloid biosynthesis [39]. In our research, the PAL was up-regulated in the 3hN/0 N, then down-regulated in the 3dN/3hN, indicating that the activity of acetylated PAL was significantly stimulated by short term N-resupply, and then the activity of PAL was reduced by long term N-resupply. So, we speculated that the activity of acetylated PAL was correlated with the synthesis of phenylpropanoids. Another important acetylated protein, DFR is dihydroflavonol 4-reductase dihydroflavonols (dihydrokaempferol, dihydroquercetin, and dihydromyricetin) to leucoanthocyanidins, which are then converted to anthocyanins, including pelargonidin, cyanidin, and delphinidin [40, 41]. In the present study, DFR was up-regulated in the 3hN/0 N, then down-regulated in the 3dN/3hN. We suggested that the activity of acetylated DFR was also stimulated by N-resupply. CHI is a key enzyme in the biosynthesis of plant flavonoids-specialized metabolites involved in diverse biotic and abiotic functions including UV protection, flower color, pollen development, root nodulation, plant architecture and chemical defenses [42]. CHI catalyzes the enantioselective formation of the tricyclic flavanone (S)- naringenin from its bicyclic precursor chalconaringenin. However, there is no report about the acetylated CHI of tea leaves in response to N nutrition. In the present study, the acetylated CHI was down-regulated under the N-resupply. We speculated that the down-regulation of CHI might reduce the synthesis of flavonoids after N-resupply. Interestingly, the naringenin 3-dioxygenase, which affected the CHI activity, was up-regulated in the 3hN/0 N, and then down-regulated in the 3dN/3hN. As for the function of the naringenin 3-dioxygenase acetylated in tea leaves had no report up to now. It is necessary to carry out in-depth research on its functions in tea plants in responding to N nutrition.

## Conclusion

In brief, this is the first extensive data on lysine acetylation in tea leaves under N-starvation/resupply. A lot of proteins related to the photosynthesis and glycolysis were found to be acetylated, including LHCA1, LHCA3, LHCB6, psaE, psaD, psaN, GAPDH, PEPC, ENL and petC. And some proteins related to flavonoids were also found to be acetylated, including PAL, DFR, naringenin 3-dioxygenase and CHI. This study not only broadens the range of metabolic processes regulated by lysine acetylation under N-starvation/resupply, but also provides a rich resource to consider the functions of lysine acetylation in tea leaves. However, more studies are needed to uncover the effects of protein acetylation in tea plants under N-treatments and to interpret the underlying mechanisms behind protein acetylation’s ability in tea leaves by sensing nitrogen nutrition.

## Additional files

Additional file 1: **Table S1.** The detailed information for reagents used in the study. (XLSX 10 kb)

Additional file 2: The more detailed information for the methods. (DOCX 21 kb)

Additional file 3: **Table S2.** The Chlorophyll fluorescence parameter (*Fv/Fm*) and N contents. (XLSX 11 kb)

Additional file 4: **Table S3.** The identified acetylated sites in tea leaves. (XLSX 303 kb)

Additional file 5: **Table S4.** The numbers of identified modification sites per protein (XLSX 35 kb)

Additional file 6: **Figure S1.** The number of proteins in the different ratio results. **(A)** the 3hN/0 N results **(B)** the 3dN/0 N results **(C)** the 3dN/3hN results. (TIF 91 kb)

Additional file 7: **Table S5.** Peptide motif. (XLSX 485 kb)

Additional file 8: **Table S6.** Motif-annot. (XLSX 91 kb)

Additional file 9: **Table S7.** Functional characterization and cellular localization of lysine acetylated proteins. (XLSX 12 kb)

Additional file 10: **Table S8.** Enrichment analysis of lysine-acetylated proteins. (XLSX 25 kb)

Additional file 11: **Table S9.** The detailed information of proteins involved in 3hN/0 N Interaction network. (XLSX 21 kb)

Additional file 12: **Table S10.** The detailed information of photosynthesis acetylated proteins involved in 3hN/0 N Interaction network. (XLSX 12 kb)

Additional file 13: **Table S11.** The detailed information of ribosome acetylated proteins involved in 3hN/0 N Interaction network. (XLSX 11 kb)

Additional file 14: **Table S12.** The detailed information of proteins involved in 3dN/0 N Interaction network. (XLSX 20 kb)

Additional file 15: **Table S13.** The detailed information of photosynthesis acetylated proteins involved in 3dN/0 N Interaction network. (XLSX 12 kb)

Additional file 16: **Table S14.** The detailed information of ribosome acetylated proteins involved in 3dN/0 N Interaction network. (XLSX 10 kb)

Additional file 17: **Table S15.** The detailed information of proteins involved in 3dN/3hN Interaction network. (XLSX 23 kb)

Additional file 18: **Table S16.** The detailed information of ribosome acetylated proteins involved in 3dN/3hN Interaction network. (XLSX 14 kb)

Additional file 19: **Table S17.** The detailed information of photosynthesis acetylated proteins involved in 3dN/3hN Interaction network. (XLSX 13 kb)

## Abbreviations

accD: acetyl-CoA carboxylase carboxyl transferase subunit beta
ALDH: aldehyde dehydrogenase
ALDO: fructose-bisphosphate aldolase
ANR: anthocyanidin reductase
CHI: chalcone isomerase
COX6B: cytochrome c oxidase subunit 6b
DFR: dihydroflavonol 4-reductase
ENL: enolase
GAPB: glyceraldehyde-3-phosphate dehydrogenase
GAPDH: glyceraldehyde 3-phosphate dehydrogenase
GLDC: glycine dehydrogenase
IDH1: isocitrate dehydrogenase
K^ac^: acetylated lysine *: a random amino acid residue
MDH: malate dehydrogenase
PAL: phenylalanine ammonia-lyase
PEPC: phosphoenolpyruvate carboxykinase
petC: Cytochrome b6-f complex
PGK: phosphoglycerate kinase
PPI: protein-protein interaction
PsaA: photosystem I P700 chlorophyll a apoprotein A1
PsaD: photosystem I subunit II
PsaE: photosystem I subunit IV
PsaN: photosystem I subunit PsaN
psbO: photosystem II oxygen-evolving enhancer protein 1
psbP: photosystem II oxygen-evolving enhancer protein 2
psbQ: photosystem II oxygen-evolving enhancer protein 3
psbS: photosystem II 22 kDa protein
PsbS: photosystem II 22 kDa protein
PTMS: post-translational modifications
rbcL: ribulose-bisphosphate carboxylase large chain
rbcS: ribulose-bisphosphate carboxylase small chain
SDS-PAGE: sodium dodecyl sulfate polyacrylamide gel electrophoresis
