# Supplementary material for: Comprehensive proteome analyses of lysine acetylation in tea leaves by sensing nitrogen nutrition
Source: BMC Genomics. 2018 Nov 26;19:840. doi: 10.1186/s12864-018-5250-4 (PMC6258439; doi:10.1186/s12864-018-5250-4)
Supplement: Supplementary file 2 — The more detailed information for the methods. (DOCX 21 kb) [file 12864_2018_5250_MOESM2_ESM.docx]

**Materials and methods**

**Plant materials**

1-year-old tea seedling of *Camellia sinensis* cv. QN3 hydroponically cultivated with the Full Nutrient (FN) solution in an air-conditioned chamber at 25±2℃/15±2℃ (16 h day /8 h night), 80±5% relative humidity and 280 μM•m^−2^•s^−1^ photon flux density 16 h light. The nutrient solutions were continuously aerated with an air pump in each hydroponic box. After 7 days the FN solution was replaced with another fresh FN solution, whereas the 0.84 mM (NH_4_)_2_SO_4_ was removed, in which the tea plants were subjected to N starvation for 7 days. After the N starvation treatment, the tea plants were harvested as 0N sample. At the same time all other tea plants were supplied with fresh nutrient solution, whereas the 0.84 mM (NH_4_)_2_SO_4_ was replaced with 2.52 mM (NH_4_)_2_SO_4_ in which plants were re-supplied with N. The tea plants were harvested at 3h and 3d after N -resupply after the harvest, the tea plants (tea leaves) were washed thrice with distilled water and finally with deionized water.

**Reagents**

All reagents unless otherwise stated were purchased from Sigma (St. Louis, America), such as Iodoacetamide (IAM), Nicotinamide (NAM) *et.al*. Acetonitrile (ACN) and TMT Kit (6 plex) were purchased from ThermoFisher Scientific (Waltham, USA). The lot number of lysine antibody agarose beads were purchased from PTM Biolabs (Hangzhou, China). And the more detail information was shown in supplement table 8.

**Leaf maximum photochemical quantum yield and Elemental analysis**

Measurement of leaf maximum photochemical quantum yield was conducted on the leaves with a portable pulse amplitude modulation fluorimeter (PAM 2000; Heinz Walz GmbH, Effeltrich, Germany). The leaf maximum photochemical quantum yield of the tea leaves in the dark was monitored for approximately 20 min at 25℃of saturating light, and the maximum quantum yield of PSII (*Fv/Fm*) was determined after the fluorescence had reached a steady level.

Samples were dried in an oven (ShangHai Fuma Test Equipment CO., LTD.). The dried samples were grinded to powder with a grinder. 0.5 g powder of each sample was put into a dry, clean PTFE vessel, and then 10 ml of nitric acid and 2 ml of perchloric acid were added and mixed. The solution was adjusted up to 25 ml with deionized water and then analyzed by inductively coupled plasma-optical emission spectroscopy (ICP-OES-Optima 8x00) (PerkinElmer, Inc., USA).

**Protein Extraction**

The samples were grinded by liquid nitrogen into cell powder and then transferred to a 5-mL centrifuge tube. After that, four volumes of lysis buffer (8 M urea, 1% Triton-100, 10 mM dithiothreitol, and 1% Protease Inhibitor Cocktail) were added to the cell powder, followed by sonication three times on ice using a high intensity ultrasonic processor (Scientz). (Note: For PTM experiments, inhibitors were also added to the lysis buffer, e.g. 3 μM TSA and 50 mM NAM for acetylation.) The remaining debris was removed by centrifugation at 20,000 g at 4 °C for 10 min. Finally, the protein was precipitated with cold 20% TCA for 2 h at -20 °C. After centrifugation at 12,000 g 4 °C for 10 min, the supernatant was discarded. The remaining precipitate was washed with cold acetone for three times. The protein was redissolved in 8 M urea and the protein concentration was determined with BCA kit according to the manufacturer’s instructions.

**Trypsin Digestion**

For digestion, the protein solution was reduced with 5 mM dithiothreitol for 30 min at 56 °C and alkylated with 11 mM iodoacetamide for 15 min at room temperature in darkness. The protein sample was then diluted by adding 100 mM TEAB to urea concentration less than 2M. Finally, trypsin was added at 1:50 trypsin-to-protein mass ratio for the first digestion overnight and 1:100 trypsin-to-protein mass ratio for a second 4 h-digestion.

**TMT Labeling**

After trypsin digestion, peptide was desalted by Strata X C18 SPE column (Phenomenex) and vacuum-dried. Peptide was reconstituted in 0.5 M TEAB and processed according to the manufacturer’s protocol for TMT kit. Briefly, one unit of TMT reagent was thawed and reconstituted in acetonitrile. The peptide mixtures were then incubated for 2 h at room temperature and pooled, desalted and dried by vacuum centrifugation. Sample 0N, 3hN and 3dN were labeled by 126,127 and 131.

**HPLC Fractionation**

The tryptic peptides were fractionated into fractions by high pH reverse-phase HPLC using Thermo Betasil C18 column (5 μm particles, 10 mm ID, 250 mm length). Briefly, peptides were first separated with a gradient of 8% to 32% acetonitrile (pH 9.0) over 60 min into 60 fractions. Then, the peptides were combined into three fractions and dried by vacuum centrifuging.

**Affinity Enrichment**

To enrich acetylated peptides, tryptic peptides dissolved in NETN buffer (100 mM NaCl, 1 mM EDTA, 50 mM Tris-HCl, 0.5% NP-40, pH 8.0) were incubated with pre-washed antibody beads (Lot number 104, PTM Bio) at 4°C overnight with gentle shaking. Then the beads were washed four times with NETN buffer and twice with H_2_O. The bound peptides were eluted from the beads with 0.1% trifluoroacetic acid. Finally, the eluted fractions were combined and vacuum-dried. For LC-MS/MS analysis, the resulting peptides were desalted with C18 ZipTips (Millipore) according to the manufacturer’s instructions.

**LC-MS/MS Analysis**

The tryptic peptides were dissolved in 0.1% formic acid (solvent A), directly loaded onto a home-made reversed-phase analytical column (15-cm length, 75 μm i.d.). The gradient was comprised of an increase from 6% to 23% solvent B (0.1% formic acid in 98% acetonitrile) over 26 min, 23% to 35% in 8 min and climbing to 80% in 3 min then holding at 80% for the last 3 min, all at a constant flow rate of 400 nL/min on an EASY-nLC 1000 UPLC system.

The peptides were subjected to NSI source followed by tandem mass spectrometry (MS/MS) in Q Exactive^TM^ Plus (Thermo) coupled online to the UPLC. The electrospray voltage applied was 2.0 kV. The m/z scan range was 350 to 1800 for full scan, and intact peptides were detected in the Orbitrap at a resolution of 70,000. Peptides were then selected for MS/MS using NCE setting as 28 and the fragments were detected in the Orbitrap at a resolution of 17,500. A data-dependent procedure that alternated between one MS scan followed by 20 MS/MS scans with 15.0s dynamic exclusion. Automatic gain control (AGC) was set at 5E4. Fixed first mass was set as 100 m/z.

**Database search**

The resulting MS/MS data were processed using Maxquant search engine (v.1.5.2.8). Tandem mass spectra were searched against tea database concatenated with reverse decoy database. Trypsin/P was specified as cleavage enzyme allowing up to 4 missing cleavages. First search range was set to 5 ppm for precursor ions, Main search range set to 5 ppm and 0.02 Da for fragment ions. Carbamidomethyl on Cys was specified as fixed modification and oxidation on Met were specified as variable modifications. FDR was adjusted to < 1% and minimum score for modified peptides was set > 40.

**Bioinformatics Methods**

Soft motif-x was used to analyze the model of sequences constituted with amino acids in specific positions of modifer-21-mers (10 amino acids upstream and downstream of the acetylation site) in all acetylated protein identified. Secondary structures of proteins were predicted by NetSurfP. The probability for one given type of secondary structure of the modified lysine residues was compared to those of the control residues for all acetylated proteins identified in this study, and *p-*value was calculated as previously described (*p*-value < 0.05). Gene Ontology (GO) annotation proteome was derived from the UniProt-GOA database ([www. http://www.ebi.ac.uk/GOA/](file:///C:\Users\BioPTM\Desktop\PTM_0078_iTRAQ_report\PTM_0078_iTRAQ_report\www.%20http:\www.ebi.ac.uk\GOA\)) and the proteins were classified by [Gene Ontology annotation](http://www.geneontology.org/) based on three categories: biological process, cellular component and molecular function. [Kyoto Encyclopedia of Genes and Genomes (KEGG)](http://www.genome.jp/kegg/) database was used to annotate protein pathway. Functional annotation tool of DAVID bioinformatics resources 6.7 was used to identify GO term and KEGG pathway. A two-tailed Fisher’s exact test was employed to test the enrichment of the protein-containing IPI entries against all IPI proteins. Correction for multiple hypothesis testing was carried out using standard false discovery rate control methods. The GO and pathway with a corrected *p*-value< 0.05 were considered significant. WoLF PSORT, a subcellular localization predication program, was used to predict subcellular localization of the identified proteins. Protein-protein interactions for the identified acetylated proteins were performed using Cytoscape software and the protein-protein interaction network was obtained from the STRING database according to the methods described previously.

**Western Blotting**

Proteins were separated by sodium dodecyl sulfate polyacrylamide gel electrophoresis (SDS-PAGE), then transferred to PVDF (Millipore) membranes and probed using anti-acetyllysine in the 1:1000 dilution (PTM Biolabs, Hangzhou, China). Secondary, the membranes were incubated in a 1:10000 dilution with horseradish peroxidase-conjugated antibody (Sigma).
