# Supplementary figures and images for: Comprehensive proteome analyses of lysine acetylation in tea leaves by sensing nitrogen nutrition
Source: BMC Genomics. 2018 Nov 26;19:840. doi: 10.1186/s12864-018-5250-4 (PMC6258439; doi:10.1186/s12864-018-5250-4)

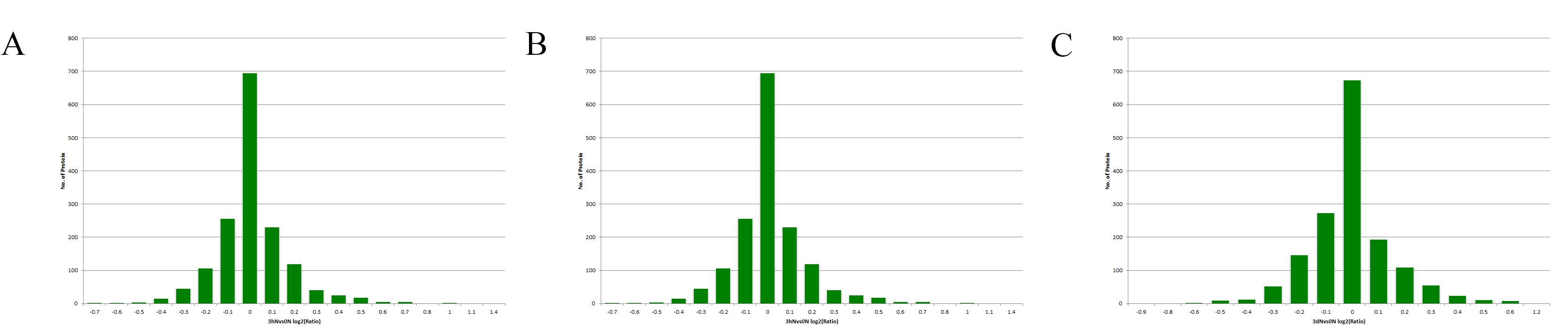

Supplement: Supplementary file 6 — Figure S1. The number of proteins in the different ratio results. (A) the 3hN/0 N results (B) the 3dN/0 N results (C) the 3dN/3hN results. (TIF 91 kb) [file 12864_2018_5250_MOESM6_ESM.tif]
